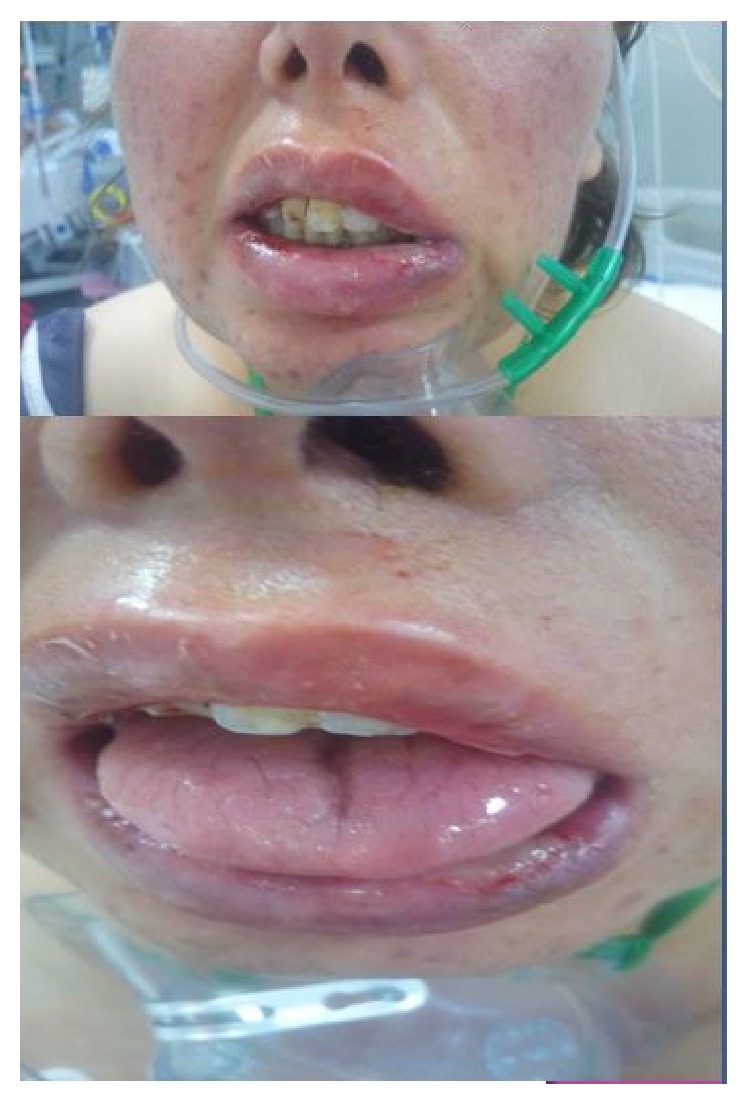# Melkersson-Rosenthal Syndrome: A Rare Cause of Recurrent Facial Nerve Palsy and Acute Respiratory Distress Syndrome

**DOI:** 10.1155/2018/1373581

**Published:** 2018-06-10

**Authors:** Behiye Deniz Kosovali, Asiye Yavuz, Fatma Irem Yesiler, Mustafa Kemal Bayar

**Affiliations:** Ankara University School of Medicine, Department of Anesthesiology and Reanimation, Division of Intensive Care Unit, Turkey

## Abstract

Melkersson-Rosenthal Syndrome (MRS) is a rare disease characterized by persistent or recurrent orofacial oedema, relapsing peripheral facial paralysis, and furrowed tongue. Pathologically, granulomatosis is responsible for oedema of face, labia, oral cavity, and facial nerve. We present a patient with MRS admitted to our hospital with acute respiratory distress syndrome (ARDS). 45-year-old woman was admitted to an emergency department with dyspnea and swelling on her hands and face. She was intubated because of ARDS and accepted to intensive care unit (ICU). After weaning from ventilatory support, peripheral facial paralysis was diagnosed and steroid treatment was added to her therapy. On dermatologic examination, oedema on her face, pustular lesions on her skin, and fissure on her tongue were detected. The patient informed us about her recurrent and spontaneous facial paralysis in previous years. According to her history and clinical findings, MRS was diagnosed.

## 1. Introduction

In intensive care units (ICU), many patients were followed up and treated for acute respiratory distress syndrome (ARDS) and this can be a life-threatening clinical issue. Most of the time, causes of ARDS are lung-related such as pneumonia, aspiration pneumonia, trauma, or systemic causes like sepsis and acute pancreatitis, and, for treatment, invasive or noninvasive mechanical ventilation (MV) is required. In this presented case report, the patient was diagnosed with ARDS but after the weaning period, she was diagnosed with Melkersson-Rosenthal Syndrome (MRS). It is an unusual presentation and ARDS and MRS may be diagnosed incidentally together. Melkersson-Rosenthal Syndrome was first described in 1928 as peripheral facial paralysis and swelling of lips. In 1931, Rosenthal completed the triad by adding the presence of fissural tongue [1].

## 2. Case Presentation

**Figure 1:**
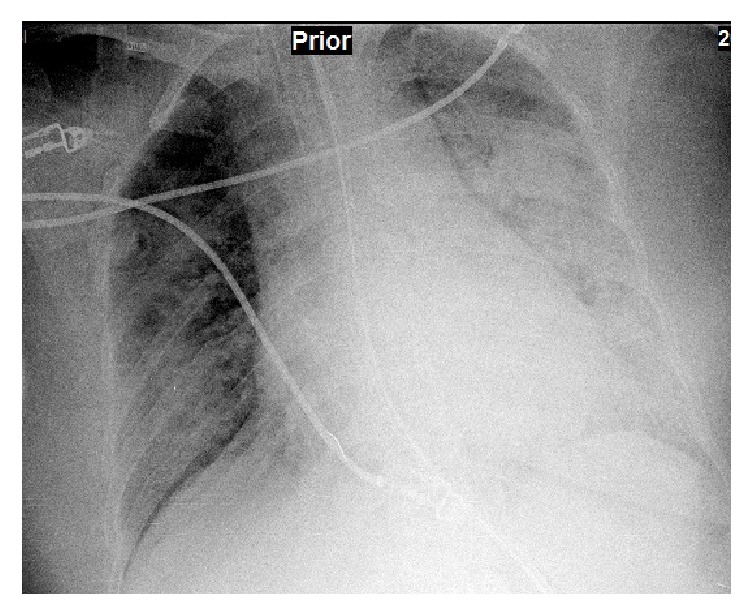


A 45-year-old woman was admitted to an emergency department with dyspnea and swelling on her hands and face for at least three days. She was nonsmoker and did not have any chronic disease. Her dyspnea and hypoxemia were getting worse and she was accepted to ICU. Noninvasive mechanical ventilation (MV) was used for initial treatment but hypoxemia was worsened; hence, she was intubated and invasive MV was used. On her physical examination, we auscultated mild crackles bilateral on lower lung zones. Her chest X-ray showed bilateral nonhomogenous infiltration at middle and lower zones (Figure 1). While initial fraction of inhaled oxygenation (FiO_2_) was 80% on MV, her PaO_2_ was 65 mmHg and lung protective MV strategies were applied. An appropriate fluid replacement, antibiotics, and other medical treatments were applied. Undergoing MV, FiO_2_ level was decreased gradually and she was weaned from MV on her fifth day of ICU stay and MV. After weaning, we observed that her oral secretions increased and her left nasolabial sulcus wiped out. On her neurological examination, abnormal findings were not found except left facial paralysis. We did not study out any pathological imagination neither on her cranial computed tomography (CT) nor on cranial magnetic resonance imaging (MRI). Peripheral facial paralysis (PFP) was diagnosed and intravenous steroid treatment 1 milligram per kilogram (methylprednisolone) was added to her therapy by neurologist. At the same time, dermatological lesion occurred and, on her dermatologic examination, oedema on her face, pustular lesions on her skin, and fissure on her tongue were detected; therefore labium mucosal biopsy was taken and mucositis was reported (Figure 2). When we talked to the patient about her symptoms, she informed us that she had recurrent and spontaneous facial paralysis in previous years. According to her medical history, signs of orofacial oedema, fissure on the tongue, and PFP, MRS was diagnosed. She was transferred from ICU to department of neurology and then she was discharged from the hospital.

## 3. Discussion

Melkersson-Rosenthal Syndrome is characterized by clinical triad, which is recurrent orofacial oedema, facial paralysis, and fissure on the tongue. It is a rare noncaseating granulomatous disease and diagnosis of MRS is a difficult issue. The etiology and mechanism of MRS are still unclear. Genetic, microbial factors such as* Mycobacterium tuberculosis* and paratuberculosis,* Borrelia burgdorferi*,* Saccharomyces cerevisiae*, and* Candida albicans*, allergic agents, and abnormal reactivity of neurovascular system may cause MRS [1]. MRS typically occurs in second and third decades of life but several patients are diagnosed after these decades because diagnosis of MRS is difficult or was not recognised previously. Incidence of MRS is estimated to be approximately 0.08% and MRS is more frequent among women [2, 3]. Our case was a woman in her fourth decade of her life. She did not have any family history and any immunological disease. Even though the clinical triad supports the diagnosis, the number of the patients who have all of these three criteria is very few. The patients are admitted to hospital with several different symptoms such as headache, paresthesia on face, epiphora, keratitis, and tongue muscle atrophy [4]. While the clinical symptoms have such a wide range, the diagnosis time should be later than onset of the symptoms. In the literature, a study reported that mean time of correct diagnosis was 9.86 years after the onset of the disease [5]. In this case report, our patient had dyspnea and swelling on her hands and face for at least three days; in addition she had recurrent facial paralysis in her medical history but she did not know when her symptoms had started. She did not know that she has ever been investigated for these symptoms by any clinician, as well. Therefore she was diagnosed at 45 years of age.

There are no specific radiological findings and diagnostic test for MRS. In this paper, the patient's cranial CT and MRI had no abnormal imagination findings, too. Diagnosis of MRS was based on clinical signs; hence, histological examination is not necessary for the diagnosis if the patient has clinical triad. The histological characteristics of MRS are granulomatous infiltrate constituted by epithelioid cells and multinucleate giant cells, without caseous necrosis, associated with some degree of lymphedema and fibrosis [6]. In 1992, Zimmer and al. reported a study that included 42 patients with MRS. Biopsy specimen was taken from swollen lips or facial tissue and 46% of patients had granulomatous inflammation, 36% had no specific inflammation, 11% had incidental findings, and 75 had no histological abnormalities [7]. Histological examination findings of our case reported mucositis. Despite this report, we did not exclude previsional diagnosis of MRS because MRS is a clinical syndrome and histopathological evidence is not necessary. The treatment involves systemic and/or topical corticosteroid. Liu and Yu treated their patients with systemic steroid and the applied minimum dose was 20 mg and maximum dose was 1000 mg in this study [5]. The initial dose was intravenous 1 mg per kg daily (80 mg) of methylprednisolone and the dose was reduced in five days and therapy was discontinued three weeks later. ARDS is characterized by acute diffuse, inflammatory lung injury, leading to increased alveolar capillary permeability and loss of aerated lung tissue. PaO_2_/FiO_2_ ratio is ≤300; bilateral opacities are seen on chest X-ray but are not fully explained by effusions, lobar or lung collapse or nodules, respiratory failure, cardiac failure, or fluid overload [8]. Clinical and radiological findings of our case were correlated with these criteria. ARDS could be classified as pulmonary or extrapulmonary ARDS. Pulmonary ARDS is related to primary pulmonary pathology such as pneumonia, aspiration pneumonia, or trauma. Although the relationship between MRS and ARDS is unknown, in the patient with MRS, among the secondary symptoms of MRS (present in more than 80% of the cases), those derived from the involvement of other cranial nerves (olfactorius, vestibulocochlearis, glossopharyngeus, hypoglossus, and trigeminus) are more frequently found. Other minor symptoms can be of neurovegetative origin such as abnormal lacrimation, profuse sweating, hemicrania, hyper- or hyposalivation, blepharospasm, nausea, vomiting, facial tics, tetanic spasm, and paresthesia at the extremes of the limbs [9]. Glossopharyngeus and hypoglossus nerves can be impressed so that deglutition is impairment and microaspiration or aspiration pneumonia can occur. We thought that our case had recurrent deglutition dysfunction when her neurological symptoms occurred and aspiration pneumonia may be the cause of ARDS. With applied lung protective MV strategies, an appropriate fluid replacement, and antibiotics during follow-up in ICU, her respiratory failure was recovered. She was transferred from ICU to department of neurology and then she was discharged from the hospital in good health.

## 4. Conclusion

MRS and ARDS togetherness is not a common issue. MRS is a rare granulomatous disease but pathological diagnosis is not always possible on the tissue specimen. The triad that is needed for clinical diagnosis is composed by recurrent PFP, orofacial oedema, and fissure on the tongue. When we review the literature, there are not any case reports with ARDS overlapping MRS. In this paper, we report the first case with ARDS overlapping MRS in the literature.

## Figures and Tables

**Figure 2:**